# Progress in Medicine

**Published:** 1896-05-23

**Authors:** 


### Progress in Medicine.

### DISEASES OF THE CIRCULATORY SYSTEM.

**The Nauheim Treatment.**—From time to time some new form of treatment is brought prominently into notice. Zealous advocacy is brought face to face with critical investigation, and between these two fires the method finds its way to a high or a low place in the therapeutic category. The Nauheim treatment for heart disease is at present under consideration. Most observers believe in its value, but differ somewhat as to the range of its applicability and the explanation of clinical facts. Dr. Saundby has given the result of his experience[1] at Nauheim, and mentions that he saw diminution of cardiac dulness in patients treated separately by the exercises and by the baths. Dr. Saundby also gives brief records of cases treated in the Birmingham General Hospital. It cannot be said that these records afford any definite evidence in favour of the treatment, but it seems to the writer of this review, who has been to Nauheim, that the cases were in all probability not of a character that would be likely to derive much benefit. Although, however, the permanent beneficial results are not very obvious, diagrams are given showing the usual reduction of cardiac dulness and alteration in the pulse that occurs after the baths and exercises. Dr. Leslie Thorne[2] also publishes cases treated in England at the baths at Llangammarch Wells, Central Wales. Four cases are given, and of these four two appeared to have been suffering from valvular lesions following rheumatism, and two had dilated hearts following influenza. All apparently showed some improvement. Dr. Herschell,[3] on the other hand, has used the treatment in four cases, and fails to see that any benefit was derived. Here, again, it may be remarked that possibly Dr. Herschell made use of cases that are not necessarily materially improved by the treatment. Dr. Herschell is entitled to doubt the efficacy of the treatment if he fail to obtain satisfactory results; but we hardly think that he is justified in saying that the only foundation for believing in the "remedial effects" of the baths and exercises is "the bare assertion of those who are working the system." Several competent observers have been to Nauheim, and some have stayed several weeks there in order to judge of the results; and few, if any, deny that the results are in great measure satisfactory. Dr. Bowles[4] and Dr. Kingscote[5] are other writers who appear to have been more fortunate than Dr. Herschell in their trials of artificially prepared baths. Dr. Bowles read a paper before the Harveian Society, describing cases of heart disease that apparently improved under the treatment, and Dr. Kingscote publishes others. The cases of Dr. Kingscote are examples of forms of heart disease that possibly are most likely to be markedly improved by the treatment. In all, seven in number, there was dilatation of the heart, but in none is there definite evidence that valvular disease was present. Iu the majority influenza was the cause of the dilatation. At Nauheim, however, dilated hearts from various other causes are seen, and when valvular disease is absent they generally more or less rapidly improve.

[1] B. M. J., 1895, Vol. II., p. 1081. [2] Lancet, 1896, Vol. I., p. 26. [3] Lancet, 1896, Vol. I., p. 413. [4] Lancet, 1896, Vol. I., p. 850. [5] Lancet, 1896, Vol. I., p. 761.

